# Removal of Pb(II) ions from aqueous solutions using natural and HDTMA-modified bentonite and kaolin clays

**DOI:** 10.1016/j.heliyon.2024.e38136

**Published:** 2024-09-19

**Authors:** Dipuo Precious Kgabi, Abayneh Ataro Ambushe

**Affiliations:** Department of Chemical Sciences, University of Johannesburg, P.O. Box 524, Auckland Park 2006, Johannesburg, South Africa

**Keywords:** Adsorption, Bentonite, HDTMA, Kaolin, Lead, Organoclays

## Abstract

This work focused on the removal of Pb(II) from aqueous solution using kaolin and bentonite clays modified with hexadecyl trimethyl ammonium bromide (HDTMA). The clays were characterized using a zetasizer, scanning electron microscopy (SEM), powder X-ray diffraction (PXRD), Brunauer-Emmet-Teller (BET), Fourier-transform infrared (FTIR) spectroscopy and thermal gravimetric analysis (TGA). Factors that influence the adsorption of Pb(II) from aqueous solution, namely pH, contact time, adsorbent mass, ionic strength, temperature and initial Pb(II) concentration were investigated. The results show that HDTMA was successfully incorporated into the kaolin and bentonite clay structures. The most favorable parameters for the adsorption of Pb(II) ions onto all adsorbents was pH of 6.0, temperature of 25 °C and adsorbent mass of 200 mg. Adsorption isotherms and kinetic studies showed that the adsorption of Pb(II) onto kaolin, bentonite and organobentonite clays followed the Langmuir isotherm and pseudo-first order kinetic model, while the adsorption onto organobentonite was better explained by the Freundlich isotherm and pseudo-second order kinetic model. Maximum monolayer adsorption capacity of organobentonite, calculated from the Langmuir model was 18.75 mg/g, which is higher than that obtained for the unmodified bentonite (14.71 mg/g); while for organokaolin it was 2.26 mg/g, which is less than that of the unmodified kaolin (4.19 mg/g). Thermodynamic studies showed that the reactions were exothermic and unfavoured at high temperatures. The adsorbents also showed good removal efficiency for up to four regeneration cycles.

## Highlights

• South African bentonite and kaolin clays were successfully modified with HDTMA.
• Adsorption onto the clays was better explained by the Langmuir and Freundlich isotherms.
• Modifying bentonite with HDTMA enhances its effectiveness in removing Pb from water.
• Bentonite and organobentonite showed the potential of serving as efficient adsorbents for the removal of Pb from water.

## 1 Introduction

With a population of over 50 million people and only about 1200 m^3^ of fresh water per person each year, South Africa is considered as one of the water scarce countries [1]. Industrial and domestic activities are some of the biggest contributors of pollutants into the South African water resources [2,3]. The pollutants that are introduced into the water systems through effluent from various industries, can be classified in two categories, namely organic (dyes, fertilizers, pesticides) and inorganic (nitrates, potentially toxic elements). This study focuses on lead (Pb) which is one of the various potentially toxic elements (PTEs) that can be found in water.

Lead is one of the common PTEs introduced into the water systems by effluent from industries [4]. Sources of lead include lead plating, batteries, and lead smelting [4,5] among others. Lead(II) when inhaled and/or ingested, can cause several ailments such as anaemia, cancer, lung insufficiency, bone lesions, renal disturbances, high-blood pressure and neurological disorders both in children and adults [[5], [6], [7]]. Lead exposure during pregnancy has also been linked with gestational pregnancy, miscarriage and premature death [8,9]. To protect the general public health from these contaminants, several organizations have set the guideline values for lead in drinking water.

The World Health Organization (WHO), the South African National Standards (SANS), and the Department of Water and Sanitation (DWS) have set maximum permissible levels (MPLs) of lead to 10 μg/L in drinking water [[10], [11], [12]], while the United States Environmental Protection Agency (US EPA) has set a permissible limit of 15 μg/L [13]. Values above this limit present health risks to both people and the environment.

Several studies have assessed the levels and health risks of lead in water bodies across South Africa. Notably, Olowoyo et al. [14] performed a risk assessment of Pb(II) in water samples from retail stores in Pretoria, Gauteng. Some samples were found to contain Pb(II) levels exceeding the WHO stipulated limit of 10 μg/L. The study indicated that the primary source of Pb(II) in drinking water is the corrosion of lead-containing materials, such as plumbing pipes used in water systems [14,15].

Elumalai et al. [1] investigated groundwater from the Mhlathuze Catchment in Kwazulu-Natal. The authors reported that Pb(II) levels exceeded safe drinking water limits at all sampled locations. High hazard quotients for all age groups indicated the water's unsuitability for drinking. The proximity to industrial and landfill sites was identified as a probable cause of contamination.

Madilonga et al. [3] reported elevated Pb(II) levels in the Mutangwi River, Limpopo province. The contamination surpassed the safety thresholds set by SANS, DWS, and WHO. Both children and adults faced carcinogenic risks from ingestion, and aquatic life was also at risk. The study stressed the need for water treatment to remove PTEs before domestic use.

These findings underscore the critical need to prevent further pollution and develop effective Pb(II) removal methods to ensure safe drinking water and to protect the health of the public. As such, the treatment of industrial and domestic effluents has become of extreme importance to ensure the levels of Pb(II) stay below the MPLs. A number of techniques have been developed to remove Pb(II) from aqueous solutions, namely chemical precipitation [16,17], ion-exchange [18,19], membrane filtration [17,20], and adsorption [20,21] among others. However, many of these techniques displayed various challenges such as high-cost of operation, the production of sludge and the usage of large amounts of chemicals [22]. Among these methods, adsorption is seen as a promising method for wastewater treatment, and has gained attention in the recent years as a preferred method for Pb(II) removal from water, due to its simplicity, low-cost, effectiveness, high efficiency, ease of implementation and operation under different conditions, low energy demands, scalability, minimal secondary pollution risks, and for continuous research-driven innovation [[23], [24], [25]]. This method offers a comprehensive and sustainable solution for addressing PTEs contamination in various water sources and applications.

One material whose characterization showed potential of serving as an adsorbent in treating water/wastewater contaminated with Pb(II) is clay material. This is due to the clay's attractive adsorption properties such as high specific surface area, high cation exchange capacity (CEC), and its availability [26,27]. Moreover, clay materials can be modified to enhance their adsorption capacities and selectivity, making them versatile tools in addressing various wastewater treatment challenges [25,28]. As a cost-effective and environmentally friendly option, clay materials offer a sustainable approach for the reduction of water pollution, and ensuring the health and well-being of people, animals and the environment.

In this study, bentonite and kaolin clays were modified with hexadecyltrimethylammonium bromide (HDTMA) to form an organoclay for the removal of Pb(II) from aqueous solutions. Thermodynamic and kinetic models were used to investigate the adsorption mechanism of Pb(II) removal onto HDTMA-modified clays. The structural changes on the clays were investigated through characterization using several techniques namely the Zetasizer, Fourier transform infrared (FTIR) spectroscopy, scanning electron microscopy (SEM), Brunauer–Emmett–Teller (BET) analysis and thermogravimetric analysis (TGA).

This study was prompted by the fact that South Africa has abundant reservoirs of bentonite and kaolin clays [29,30], yet there has been limited studies done on the application of these clays as adsorbents for the removal of PTEs from water/wastewater. Furthermore, many studies have reported on the usage of organically modified clays for the removal of organic contaminants [28,[31], [32], [33]], however, there is still limited studies on their usage in removing inorganic contaminants such as Pb(II) from water. As such, this study is significant in that it investigates the potential of bentonite, and kaolin clays and their organically modified forms as adsorbents for removing Pb(II) from aqueous solutions. To the best of authors knowledge, there has not been any reported studies of the usage of HDTMA-modified South African bentonite and kaolin clays for the adsorption of Pb(II) from aqueous solutions. Thus, the use of South African clays that are abundantly available to solve the domestic problems of water pollution by PTEs such as Pb(II) is imperative and will have significant contribution to the protection of the environment.

## 2 Methods and materials

### 2.1 Instrumentation and reagents

Only analytical grade chemicals were used in this study, the chemicals used were lead nitrate (Pb(NO_3_)_2_), hexadecyl trimethyl ammonium bromide (HDTMA), nitric acid (HNO_3_, 65 %), sodium hydroxide (NaOH, 98 %), all purchased from Sigma-Aldrich (Darmstadt, Germany); hydrochloric acid (HCl, 37 %) purchased from Chemistore (Kempton Park, South Africa); as well as silver nitrate (AgNO_3_) purchased from Rochelle Chemicals (Johannesburg, South Africa). All reagents were used without further purification. Natural kaolin (CEC = 13.1 meq/100 g) and bentonite (78.6 meq/100 g) clays were purchased from Micronized (Johannesburg, South Africa) and Imerys (Pretoria, South Africa), respectively. The flame atomic absorption spectrometer AAS-7000 from Shimadzu (Kyoto, Japan) was used to measure the concentrations of Pb(II) in solutions before and after adsorption. The surface morphology of the clays was investigated using the TESCAN VEGA 3 LMH SEM (TESCAN, Brno-Kohoutovice, Czech Republic); the zeta potential of the clays was determined using the Malvern Zetasizer (Malvern Panalytical, Randburg, South Africa). The functional groups present were identified by means of an IR Affinity-1S FTIR spectrophotometer (Shimadzu, Kyoto, Japan). An STA 6000 TGA (TA Instruments, New Castle, DE, USA) was used to determine the thermal stability of the clays. Mircromeritics ASAP 2020 Gas Absorption BET (Micrometrics, Brussels, Belgium) was used to determine the pore size, pore volume and specific surface areas of the clays. The interlayer space as well as the phase composition and crystal structure of the clays were determined by means of the PANanalytical Philips X'Pert PXRD (PANanalytical, Almelo, The Netherlands). The Milli-Q water (resistivity of 18 MΩ cm) used to prepare solutions was obtained from Milli-Q® Direct 8/16 System (Merck, Darmstadt, Germany). The solutions were stirred on an RS Pro stirrer plate (RS Components, Midrand, South Africa).

### 2.2 Characterization of the natural and modified clays

The clays were characterized for their physicochemical properties using various techniques, namely SEM, PXRD, BET, TGA, and the zetasizer. The operational conditions, sample preparations and characterization procedures are reported in previously published work [26].

### 2.3 Preparation of the organoclays

A mass of 30.0 g of the clay samples were annealed at 500 °C to remove any organic material and increase the clay's stability [34]. After annealing, the organoclays were prepared according to a procedure reported in the literature [35]. A mass of 20.0 g of each clay was dispersed in 1500 mL Millli-Q water, the solution was stirred with high speed stirrer for 2 h until the clay was homogenously dispersed in the water. Hexadecyl trimethyl ammonium bromide was weighed (based on the clay's 0.5 × CEC) and dissolved in 500 mL Milli-Q water until completely dissolved. The amount of HDTMA used was calculated based on the CEC of the clay as follows:

(1) WeightofHDTMA(g)=nxCECxAxB

where A = weight of the clay (g) and B = is molecular weight of HDTMA (g/mol); n = ratio to be used (0.5). The amounts of HDTMA used for the surface modification, calculated based on equation (1) is given in Table 1.

**Table 1:** Amount of HDTMA calculated for organoclay preparation.

Clay	Weight of clay (A) (g)	Molecular weight of HDTMA (B) (g/mol)	Ratio used (n)	Clay's CEC (meq/100 g)	Amount of HDTMA required (g)
Bentonite	20.0	364.45	0.5	78.6	**2.86**
Kaolin	20.0	364.45	0.5	13.1	**0.48**

The dissolved HDTMA solution was poured into the clay's solution and stirred for 24 h at room temperature. The mixed solution was then precipitated at 4000 rpm in a centrifuge. The precipitate was washed several times with Milli-Q water until it was free of bromide ions (tested with 0.1 M AgNO_3_ solution). The resulting precipitate was then dried overnight in an oven at 60 °C. The dried organoclay samples were then ground using a pestle and mortar, sieved through a 100 μm sieve, and stored in a desiccator until use.

### 2.4 Batch adsorption studies

Batch adsorption studies were performed through stirring the solutions on a magnetic stirrer plate at a speed of 300 rpm. The effects of pH (2–6), contact time (30–300 min), temperature (25–70 °C), adsorbent mass (0.05–0.3 g), initial Pb(II) concentrations (5–100 mg/L) and the concentration of KCl (0.0–0.5 mol/L) on the adsorption were investigated. The adsorption studies were done in triplicates.

The percentage removal (%) and adsorption capacity Q_e_ (mg/g) were calculated using equations described by Castro-Castro et al. [36]:

(2) $$Qe=\frac{Co-Ce}{m}x\;V$$

(3) $$\%\;removal=\frac{Co-Ce}{Co}\;x\;100$$

where Q_e_ (mg/g) is the adsorption capacity, m is the adsorbent mass (g), V is the volume (L) of the solution, C_o_ (mg/L) is the Pb(II)concentration before adsorption, and C_e_ (mg/L) is the Pb(II) concentration after adsorption.

### 2.5 Analytical figures of merit

To assess the reliability and accuracy of the method used in the quantification of the Pb through FAAS, various analytical figures of merit were determined, the percentage recovery experiments were also conducted. The linearity of the method was determined by analysing the standard solutions of Pb in the range of 0.5–5 mg/L and then determining the coefficient of determination (R^2^). The limit of detection (LOD) and limit of quantification (LOQ) were determined using reagent blanks. The concentration of 10 reagent blank solutions was determined, and the LOD and LOQ were calculated as three times and ten times the standard deviation of the average of ten individually prepared reagent blank solutions. To determine the precision and repeatability of the method, the percentage relative standard deviation (%RSD) was determined. To assess the accuracy of the total concentration of Pb determined in the sample solutions, the river water sample with a known concentration was spiked with Pb at 1XLOQ and 10XLOQ levels and the percentage recoveries were calculated. The accuracy of the method was validated by the percentage recoveries obtained. All the experiments and analysis were performed in triplicates.

### 2.6 Real water application and regeneration studies

Borehole water was used to study the applicability of the clay adsorbents in removing Pb(II) from real water samples as well as for the regeneration-reuse studies. The concentration of Pb(II) in borehole water was significantly lower than the highest Pb concentration that can be removed by the adsorbents, as such, the borehole water was spiked with 30 mg/L of Pb for adsorption onto bentonite and organobentonite, as well as 10 mg/L of Pb for adsorption onto kaolin and organokaolin. The usage of borehole water ensures the presence of matrix effect and as such can be used as a real water sample. The experiments were all performed in triplicates at a solution pH of 6, and adsorbent mass of 1.0 g at room temperature. The best contact times for adsorption onto bentonite, kaolin and organokaolin was 120 min, and 300 min for organobentonite. After adsorption, the clay and Pb solution mixtures were filtered through a 0.45 μm filter papers and the clear solution was analyzed using FAAS.

To regenerate the spent adsorbents, the residues, i.e., the clay-adsorbed materials were agitated in 100 mL of 0.01 M HCl solution for 60 min. After agitation, the mixture was filtered and the obtained residue was rinsed with Milli-Q water and oven dried overnight at 60 °C [37], ground to a fine powder using a pestle and mortar and sieved through a 100 μm sieve. The recovered clays were used again to treat the real water samples. The regeneration-reuse process was repeated 3 more times.

### 2.7 Adsorption isotherms

Three mostly used isotherm models (Langmuir, Freundlich and Dubinin-Radushkevich models) were used to describe the adsorption nature of Pb(II) onto the adsorbents. The Langmuir is associated with monolayered adsorption, Freundlich model is related to the multi-layered adsorption [38], while the Dubinin-Radushkevich model is based on the assumption of a uniform adsorption potential at the surface [39]. The non-linear equations of the models are as follows: the non-linear equation of the Langmuir model is [38]:

(4) $$q_{e}=\frac{q_{m}K_{L}C_{e}}{1+K_{L}C_{e}}$$

where q_m_ is the maximum monolayer adsorption capacity (mg/g) and K_L_ is the Langmuir equilibrium constant (l/mg). The Freundlich model is related to the multi-layered adsorption, and the equation of the model is [38]:

(5) $$q_{e}=K_{f}C_{e}^{1/n}$$

where K_F_ is the adsorption capacity when Pb(II) ion equilibrium concentration is 1, and n is the degree of adsorption dependency on the equilibrium concentration [(mg/g)/(mg/L)]. The Dubinin-Radushkevich model is based on the assumption of a uniform adsorption potential at the surface, its non-linearized equation is [39]:

(6) $$qe=q_{DR}\;\exp\left(-B_{DR}\varepsilon^{2}\right)$$

ε is the Polanyi potential (kJ/mol) and can be calculated as per this equation:

(7) $$\varepsilon =\text{RTln}\left(1+\frac{1}{C_{e}}\right)$$

where R is the universal gas constant (8.314 J mol^−1^ K^−1^) and T is the temperature (K), the mean free sorption energy E (kJ.mol^−1^) was calculated using the value of B (equation (6)) and computed using the following equation [39]:

(8) $$E=\frac{1}{\sqrt{2B}}$$

### 2.8 Kinetic models

The effect of contact time on the removal of Pb(II) ions from solution was investigated using three different kinetic models to show the nature of the adsorption process as well as the rate limiting process. The models applied are namely pseudo-first order, pseudo-second order and intra-particle-diffusion model. The non-linearized forms of the models as reported in the literature [25,29,40] are given below:

(9) $$\text{Pseudo}-1^{\text{st}}\;\text{order}:q_{t}=q_{e}\left(1-e^{-K1t}\right)$$

(10) $$\text{Pseudo}-2^{\text{nd}}\;\text{order}:q_{t}=\frac{K_{2}q_{e}^{2}t}{1+k_{2}q_{e}t}$$

(11) $$\text{Intraparticle}-\text{diffusion}:q_{t}=k_{id}t^{1/2}+c_{i}$$

where *k*_*1*_, *k*_*2*_ and *k*_*id*_ are the pseudo-1st order, pseudo-2nd order and intra-particle diffusion adsorption rate coefficients, *q*_*e*_ and *q*_*t*_ are the values of the amount of Pb(II) adsorbed per unit mass at equilibrium and at time *t*, respectively.

### 2.9 Thermodynamic studies

The possible adsorption mechanisms were verified through carrying out thermodynamic experiments at different temperatures. The thermodynamic equations used, as described elsewhere [[41], [42], [43]] are:

(12) $$\ln\;K_{d}=\frac{\Delta S}{R}-\frac{\Delta H}{RT}$$

where $K_{d}$ is the distribution coefficient (q_e_/C_e_), ΔH is the change in enthalpy (kJ.mol^−1^), ΔS is the change in entropy (J.K.mol^−1^), T is the temperature (K) and R is the universal gas constant (8.314 J mol^−1^ K^−1^). The change in Gibbs energy ΔG (kJ.mol^−1^) was determined using the following equation:

(13) ΔG=ΔH−TΔS

The values of ΔH and ΔS for Pb(II) adsorption were calculated by fitting the experimental data to Equation (12) while the ΔG values were obtained using Equation (13).

### 2.10 The FAAS operational conditions

The instrumental conditions used for the analysis of Pb(II) by the FAAS are reported in Table 2.

**Table 2:** FAAS operating conditions for Pb(II) analysis.

Parameter	Settings
Hollow cathode lamp	Pb
Current	10 mA
Slit Width	0.7 nm
Wavelength	283.3 nm
Acetylene flow rate	1.6 L/min
Air flow rate	15 L/min
Sample flow rate	5 mL/min
Background correction	Deuterium lamp

## 3 Results and discussion

### 3.1 Characterization

To confirm the successful modification of the natural clays with HDTMA, several characterization techniques were employed, and the findings are discussed here.

#### 3.1.1 SEM characterization

**Fig. 1:**
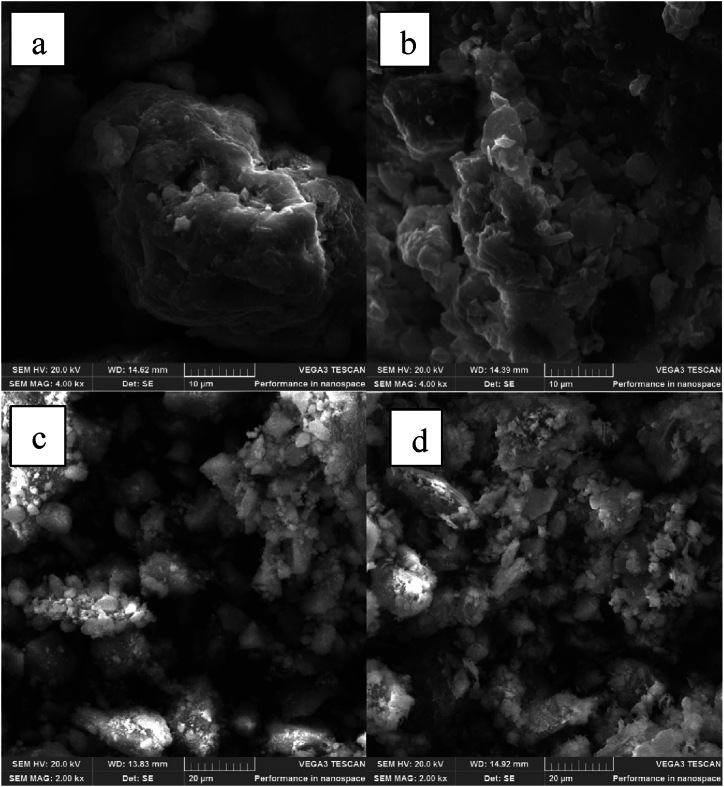
SEM images of (a) bentonite (b) organobentonite (c) kaolin and (d) organokaolin.

Scanning electron microscopic images of the modified and unmodified clays are depicted in Fig. 1. Bentonite (a) displays sponge-like structures which are separated by distinct void spaces, however, upon modification with HDTMA, there is aggregation on the surface of the clay structure. The void spaces between the clay particles on bentonite also diminish after modification. The modification of bentonite by HDTMA changes the morphology greatly, and similar observations were reported in other studies [25,44]. The SEM images of kaolin (c) shows agglomerated flake-like structure with void spaces also appearing between the particles, the surface morphology of kaolin upon modification does not change drastically, except the void spaces diminish and there is more particle agglomeration observed. A study by Prabawa et al. [45] also reported no significant changes in the morphology of kaolin upon modification with urea, however, the study reported a slight increase in the distance between the kaolin particles upon modification, which is opposite to what was observed in the current study.

#### 3.1.2 Characterization using BET, PXRD and Zetasizer

Summary of the BET, PXRD and Zetasizer characterization results of the four adsorbents are shown in Table 3.

**Table 3:** Summary of BET, PXRD and Zetasizer results.

Parameters	Adsorbent			
	Bentonite	Organobentonite	Kaolin	Organokaolin
BET specific surface area (m^2^/g)	38.7	19.4	9.51	8.37
Pore size (Å)	190.9	123.9	957.6	95.12
Pore volume (cm^3^/g)	0.185	0.0191	0.228	0.0251
Point of zero charge (pHpzc)	None	7.6	2.5	11.1
d_001_-space (Å)	17.6	17.9	3.59	3.68

The BET specific surface area results indicate that the modification of bentonite and kaolin by HDTMA reduced the specific surface area by almost 50 % and 12 %, respectively. The average pore sizes and pore volumes also decreased upon modification. A study by Andrunik and Bajda (2019) [46] also reported a decrease in pore volume and specific surface area of bentonite clay upon modification with a cationic surfactant. The decrease in pore size, pore volume and specific surface area can be explained by the change in surface properties of the clays upon incorporating HDTMA. The addition of HDTMA into the interlayer space causes an expansion of the interlayers. The d_001_ space for bentonite increased from 17.6 to 17.9 Å, while that of kaolin increased from 3.59 to 3.68 Å upon modification. This behaviour leads to an increase in the packing density of the organoclays, as a result, the pores get blocked leading to a decrease in pore size, pore volume and BET specific surface area [47,48].

#### 3.1.3 Characterization using FTIR spectroscopy and TGA

**Fig. 2:**
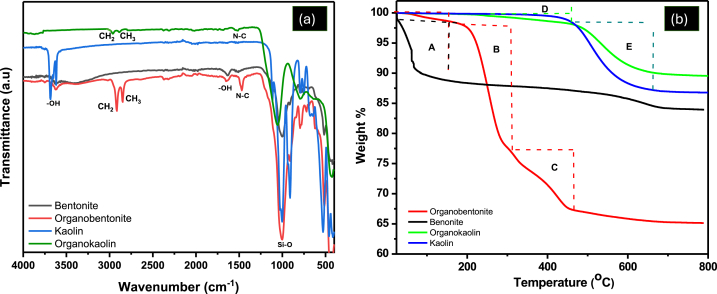
(a) FTIR spectra and (b) TGA curves of the natural and modified kaolin and bentonite clays.

To confirm the successful incorporation of HDTMA into the clays, further characterization of the modified clay samples was conducted by means of FTIR spectroscopy and TGA analysis. The results of the analysis are recorded in Fig. 2. The FTIR spectra and TGA thermograms of the unmodified kaolin and bentonite clays used in this study are discussed in previous work [26]. The successful intercalation of HDTMA into the clays’ structures has been confirmed in other studies by the appearance of CH_2_ and CH_3_ group peaks in the region around 2930 and 2870 cm^−1^, respectively [25,44]. This study also confirmed the successful modification of the clays by HDTMA through these characteristic peak intensities. The sharp CH_2_ and CH_3_ vibrations observed at 2933 and 2864 cm^−1^ confirm the intercalation of HDTMA onto the clays. The spectra of bentonite, kaolin and organobentonite also show the presence of -OH stretching bands at 3618–3684 cm^−1^. The bands at 995–1003 cm^−1^ are assigned to the Si-O stretching vibrations [26,49] The band at 1472 cm^−1^ is attributed to the N-C stretching of HDTMA [36], further confirming the successful modification.

To further reconfirm this intercalation of HDTMA into the structures of the clays, the clays were subjected to thermal treatment using TGA set to a temperature range of 25–800 °C for 90 min. The TGA curve of organobentonite shows three stages of mass loss. The first stage (A) at 90–200 °C shows a mass loss of 2.8 % which is attributed to dehydration of physically adsorbed water molecules and those intercalated in the interlayers. This mass loss is lower than that of unmodified bentonite (12.23 %) and confirms the modified bentonite is less hydrophilic [50]. The second stage (B) occurs between 200 and 300 °C shows a mass loss of 19.2 % which is due to the decomposition of HDTMA on the surface of bentonite [51]. The mass loss of 9.7 % occurring at the third stage (C) between 300 and 450 °C is due to the decomposition of HDTMA in the interlayer spaces of the clay [50,51].

The TGA curve of organokaolin shows two stages of mass loss, the first stage (D) at temperatures between 220 and 450 °C is likely due to the decomposition of both the HDTMA on the surface and the interlayers of the clay. This mass loss is very minimal compared to that of organobentonite, this is because kaolin has less CEC, as such less HDTMA was used to modify the clay. The second stage of mass loss (E) at 450–650 °C is due to dehydroxylation [26]. The mass loss due to dehydroxylation is less in organokaolin (7.11 %) compared to kaolin (13.37 %). The annealing of the kaolin prior to modification with HDTMA dehydrolyzes it [52], as such organokaolin has less OH groups in its inter layers resulting in less dehydroxylation [52]. Both the FTIR spectra and TGA results confirm the successful intercalation of HDTMA onto bentonite and kaolin clays.

### 3.2 Factors affecting the Pb(II) uptake

#### 3.2.1 Effect of pH

**Fig. 3:**
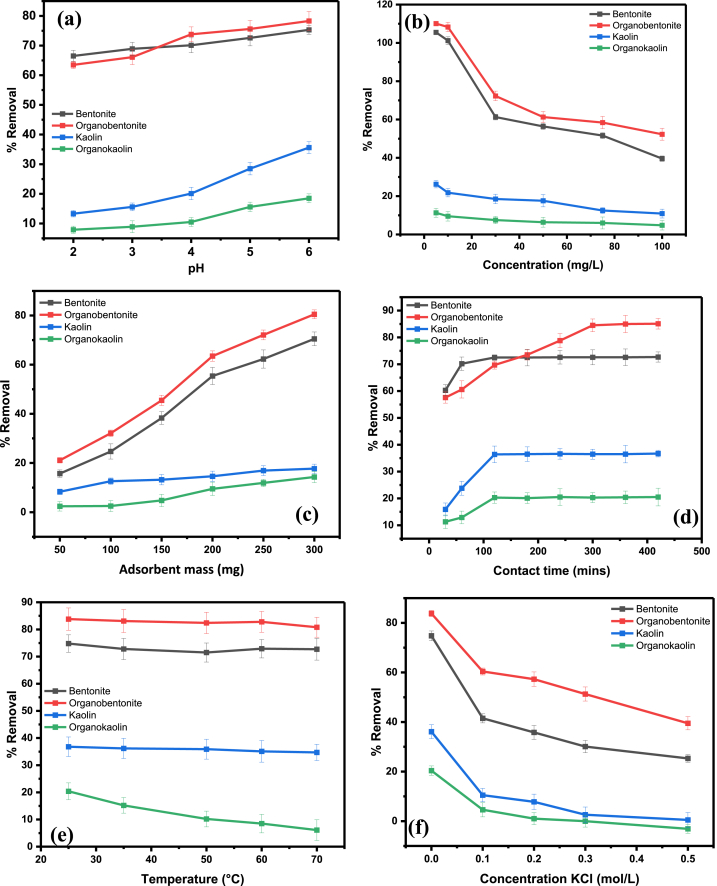
The effects of (a) pH, (b) initial Pb(II) concentration, (c) adsorbent mass, (d) contact time, (e) temperature and (f) KCl ionic strength (KCl concentration) on the removal of Pb(II) from solution.

The effect of solution pH on the removal of Pb(II) using bentonite and HDTMA-bentonite was studied through adsorbing 10 mg/L of Pb(II) solution in 100 mL at pH range of 1–6. The mass of clay used was 300 mg, and the solution was agitated at 300 rpm for 2 h. Fig. 3a shows the results of the study. The pH plays an important role in the removal of Pb(II) due to its impact on the formation of different forms of Pb(II) compounds such as Pb(OH)^+^ or Pb(OH)_2_ precipitates [53]. As depicted in Fig. 3a, the effectiveness of the adsorption of Pb(II) onto all four adsorbents increased as the pH was raised from 1 to 6. Above the pH of 6, Pb(II) ions precipitated out of solution, hence pH 6 was chosen as the ideal pH for this study. The precipitation of Pb(II) ions from solution at pHs above 6 was also reported in other studies [54]. Bentonite exhibits a negatively charged surface throughout all pH ranges, as determined by the zeta potential studies. This allows it to remove Pb(II) ions at most pH ranges. The adsorption phenomenon of bentonite and kaolin can be well explained by ion exchange mechanism [25,39]. However, the maximum removal of Pb(II) cations at pH of 6 by the two organoclays indicates that the main mechanism of removal is not ion exchange since the point of zero charge (pH_PZC_) is at pH 7.8 and 10.2 for organobentonite and organokaolin, respectively. This finding suggests the removal of Pb(II) ions by the organoclays is likely to be due to the formation of inner-sphere complexes, as reported in a similar study [25]. The modification of bentonite and kaolin reduces hydophilicity of the clay's structures, this allows for the Pb(II) ions to form inner-sphere complexes with the silanol and aluminol groups on the surface of the organoclays [55].

#### 3.2.2 Effect of Pb(II) concentration

To investigate the maximum Pb(II) concentration removal by the adsorbents, the initial metal ion concentration was varied from 5 to 100 mg/L. The experiments were performed at a pH of 6. Fig. 3b shows the impact of initial Pb(II) concentrations on the adsorption efficiency of the adsorbents. The results clearly show the removal efficiency decreases with an increase in initial concentration for all adsorbents. This can be attributed to adsorption site saturation [25]. As the concentration of Pb(II) ions in the solution increase, the available adsorption sites on the clay start to get saturated. At higher concentrations, more and more of these sites become saturated with Pb(II) ions, leading to reduced capacity for further adsorption [56,57]. The maximum adsorption concentration for bentonite and organobentonite was 30 mg/L, while for kaolin and organokaolin, it was 10 mg/L. As expected from the elemental composition of the clays, natural kaolin showed a lower Pb(II) removal compared to natural bentonite due to it having low CEC and specific surface area.

The presence of cations such as Na^+^, K^+^ and Ca^2+^ allows for bentonite's better ion-exchange capacity [58]. Modified kaolin showed a lower Pb(II) removal compared to the natural kaolin, which can be attributed to the increased hydrophobicity of the clay. Because kaolin has a low CEC, the incorporation of HDTMA into its structure renders it almost completely hydrophobic, making it difficult to adsorb the Pb(II) ions in solution.

#### 3.2.3 The effect of adsorbent mass

The effect of adsorbent mass on the removal of Pb(II) was studied through adsorbing 10 mg/L of Pb(II) solutions using kaolin and organokaolin, as well as 30 mg/L of Pb(II) solutions using bentonite and organobentonite at pH of 6 for 2 h. The mass of the adsorbents was varied between 50 mg and 300 mg. Fig. 3c shows the results of the study. As expected, the percentage removal of Pb(II) ions from solution increased with an increase in the adsorbent mass. This is because the addition of more adsorbent implies an increase in the available adsorbent sites for adsorption to take place [42]. An increase in the specific surface area allows more opportunities for Pb(II) ions to interact with the surface of the clay with less competition. For bentonite and organobentonite, the mass of 200 mg was sufficient to remove 55 % and 65 % of the 30 mg/L Pb(II) ions. Although the percentage removal for kaolin and bentonite increased with an increase in the adsorbent dosage, beyond 200 mg/L there was little change in percentage removal, thus, 200 mg was chosen as the ideal amount of adsorbent for this study.

#### 3.2.4 The effect of contact time

To study the effect of contact time, solutions of 10 mg/L of Pb(II) were treated with kaolin and organokaolin, while 30 mg/L solutions were treated with bentonite and organobentonite using 200 mg of adsorbent at pH 6. The contact time was varied from 30 to 400 min. The results from Fig. 3d indicated that adsorption efficiency increased with contact time until equilibrium: 180 min for bentonite, 120 min for kaolin and organokaolin, and 300 min for organobentonite.

Within the first 30 min, over 50 % of Pb(II) was adsorbed by bentonite and organobentonite, compared to approximately 15 % and 10 % by kaolin and organokaolin, respectively. The removal capacity increased steadily until equilibrium. Organobentonite's Pb(II) removal gradually increased until equilibrium at 300 min. The modification with the surfactant facilitates inner-sphere complex formation [59]. This formation of inner-sphere complexes, while increasing the removal efficiency, also prolonged the time to reach equilibrium due to complex interactions [25,28]. The highest percentage removals were 20.1 % for organokaolin, 36.4 % for kaolin, 72.7 % for bentonite, and 82.5 % for organobentonite.

#### 3.2.5 The effect of temperature

The effect of temperature on the removal of Pb(II) was studied through adsorbing 10 mg/L of Pb(II) solutions using kaolin and organokaolin, as well as 30 mg/L of Pb(II) solutions using bentonite and organobentonite at pH of 6, using 200 mg of the adsorbent at the equilibrium contact times reported in section 3.2.4. The temperature was varied between room temperature (23–25 °C) and 70 °C. The findings are shown in Fig. 3e. The results show that Pb(II) removal efficiency by the four adsorbents decreased with an increase in temperature, indicating an exothermic nature of the adsorption. The decrease in the percentage removal could be attributed to the weakening of the attraction between the Pb(II) ions and the clay material [60,61]. This results in a decrease in sorption of the ions. At high temperatures the boundary layer's thickness is decreased because of the increased tendency of the Pb(II) ions to desorb from the surface of the adsorbent back into the solution, which results in a decreased adsorption as the temperature is increased [60]. Thus, room temperature was chosen as the best temperature for this study. The final concentrations of Pb(II) in solution after treatment were reduced from 10 mg/L to 7.96 mg/L using organokaolin and to 6.33 mg/L using kaolin. For bentonite and organobentonite, the remaining Pb(II) ions in solution were reduced from 30 mg/L to 8.12 mg/L and 5.25 mg/L, respectively.

#### 3.2.6 The effect of ionic strength (KCl concentration)

The effect of ionic strength on the adsorption of Pb(II) ions onto the various adsorbents was studied by varying KCl concentration. The results shown in Fig. 3f demonstrated that the removal efficiency of Pb(II) decreased with increasing KCl concentration. Specifically, organobentonite's adsorption dropped from 83 % to 41 %, while bentonite, kaolin, and organokaolin showed reductions from 78.2 %, 37.4 %, and 20.8 %–21.1 %, 3.7 %, and 0.1 %, respectively. The electrostatic attraction between the K^+^ cations and the permanent negative charge on the clay's surface can result in the formation of outer-sphere complexes at the planar sites. Inner-sphere complexes can also be formed between the K^+^ cations and the Al-O- and Si–O–groups in the clays' structure [25]. Thus, this leads to fewer adsorption sites being available for the uptake of the Pb(II) ions, resulting in lower percentage removal of Pb(II) by the adsorbents. Similar findings were reported by Ref. [62], where the presence of Na^+^ in solution resulted in a decrease in the adsorption of Cd(II) ions.

### 3.3 Analytical figures of merit

Validation of the analytical method was done through determining the usual analytical figures of merit, namely the linearity, precision, accuracy, as well as the LOD and LOQ (see Table 4).

**Table 4:** Analytical figures of merit for the method.

R^2^	LOD (mg/L)	LOQ (mg/L)	% RSD
0.9996	0.036	0.120	1.2

The method was linear as proven by the R^2^ value of 0.9996. The calculated %RSD was 1.2 % indicating high precision and repeatability of the quantitative analysis [63]. These results confirm the reliability of the method for quantifying Pb(II) in water. The calculated percentage recoveries obtained are recorded in Table 5, and the method showed to give high percentage recoveries of 85.5 and 88.4 % at 1XLOQ and 10XLOQ spiking levels, respectively. These percentage recoveries fall within the acceptable range of 85–115 % [64], and confirm the accuracy of the method [62].

**Table 5:** Percentage recoveries at 1XLOQ and 10XLOQ level.

	Unspiked sample (mg/L)	Spiking concentration (mg/L)	Concentration after spiking (mg/L)	Percentage recovery (%)
1XLOQ	0.136	0.120	0.219	85.5
10XLOQ	0.136	1.20	1.049	86.8

### 3.4 Adsorption isotherms

**Fig. 4:**
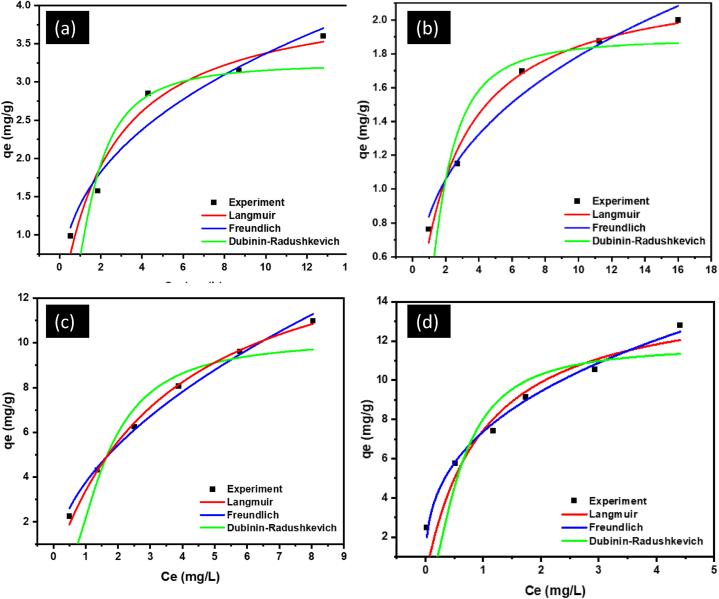
Non-linear adsorption isotherm models for the uptake of Pb(II) onto (a) kaolin (b) organokaolin (c) bentonite and (d) organobentonite at room temperature.

To determine the optimal adsorption equilibrium correlation for the uptake of Pb(II) onto the natural and modified clays at room temperature, non-linear isotherm models were analyzed using OriginPro 8.5 software. According to literature, non-linear regression is the most practical and precise method for estimating the parameters of the isothermal models because it employs the original equations rather than modified versions, which could introduce bias into the results [65]. The plots of the non-linear isotherms of Pb(II) are shown in Fig. 4 and the adsorption parameters are given in Table 6.

**Table 6:** Non-linear isotherm parameters for the adsorption of Pb(II) onto the natural and modified clay adsorbents.

Model Parameter	Adsorbent			
	Bentonite	Organobentonite	Kaolin	Organokaolin
Langmuir				
K_L_ (L/mg)	0.2743	1.0247	0.4149	0.4447
q_m_ (mg/g)	14.713	18.752	4.1907	2.2591
R^2^	0.9965	0.9072	0.9659	0.9875
RMSE	0.4377	2.485	0.4078	0.1172
χ^2^	0.0479	1.544	0.0554	0.0046
Freundlich				
K_F_(mg^(1-1/n)^1^1/n^/g)	3.770	7.380	1.3980	0.8432
n	1.90	2.82	2.61	3.07
R^2^	0.9926	0.9913	0.9531	0.9705
RMSE	0.6325	0.7632	0.4781	0.1799
χ^2^	0.1000	0.1456	0.0762	0.0108
Dubinin-Radushkevich(D-R)				
q_m_	10.139	11.719	3.244	1.8885
β (mol^2^kJ^2^)	0.07561	0.05472	0.9426	0.20604
E (kJ/mol)	4.775	67.61	3.128	3.395
R^2^	0.9462	0.9576	0.7483	0.9040
RMSE	30.711	17.979	10.750	3.028
χ^2^	0.9466	0.3242	0.1159	0.0092

The calculated isotherm parameters inform us about the nature and type of adsorption. The adsorption of Pb(II) onto bentonite, kaolin and organobentonite was better explained by the Langmuir model since it gave the highest linear regression coefficient (R^2^) values of 0.9965, 0.9659, and 0.9875, respectively. The model also gave the smallest root mean square errors (RMSE) of 0.4377, 0.4078, and 0.1172; as well lowest chi-squared errors (χ^2^) of 0.0479, 0.554, and 0.0046, respectively. According to Tonk et al. [65], the best fit is attained from a model with highest R^2^, lowest RMSE and χ^2^ values, since the data indicates a good fit between the experimental data and the theoretical model. Based on the theoretical properties of the Langmuir isotherm, the adsorption occurs on a homogenous surface, forming a monolayer of adsorption [39,66]. The mean free-energies (E) of adsorption onto bentonite, kaolin and organokaolin (as calculated from the D-R isotherm) is 4.775, 3.128, and 3.395 kJ/mol, respectively. These E values are less than 8 kJ/mol, indicative of physical adsorption [65].

The adsorption of Pb(II) onto organobentonite was better fitted onto the Freudlich isotherm model since the model gave the highest R^2^ (0.9913), lowest RMSE (0.736) and χ^2^ (0.1456) values. These results demonstrate that the adsorption occurs in a multilayer fashion, on a heterogenous surface [38,66]. The change in the adsorption behaviour of the natural and modified bentonite clays can be explained by the difference in adsorption mechanism of the natural clay against the organoclays. Natural clay primarily rely on ion-exchange for adsorption, leading to monolayer adsorption, while the introduction of HDTMA onto the clay introduces other chemical adsorption modes such as the complexation of Pb(II) ions with the functional groups on the surface of the clay [67,68]. These chemical interactions can lead to the formation of multilayer adsorption as multiple Pb(II) ions form bonds with the modified surface. The value of E for the adsorption of Pb(II) onto organobentonite is 67 kJ/mol, indicating a highly stronger interaction, thus, chemisorption in addition to ion-exchange, can be said to be the main mechanism of removal [69]. According to Ismail et al. [70], chemisorption processes involve bonding energies greater than 40 kJ/mol.

The dimensionless constant *n* calculated from the Freundlich model yielded a value greater than 1 for all adsorbents. This value falls within the range of 1–10 signifying that the adsorption of Pb(II) onto the clays is mostly favorable [67,71]. The Langmuir maximum monolayer adsorption capacity for modified bentonite clay is 18.752 mg/g, which is higher than that obtained for the unmodified sample (14.713 mg/g), while for the modified kaolin clay is 2.259 mg/g which is lower than the one for the unmodified kaolin (4.191 mg/g). This highlights that the incorporation of HDTMA into kaolin decreases its Pb(II) removal efficiency, while the opposite is true for bentonite modified with HDTMA.

### 3.5 Kinetic studies

**Fig. 5:**
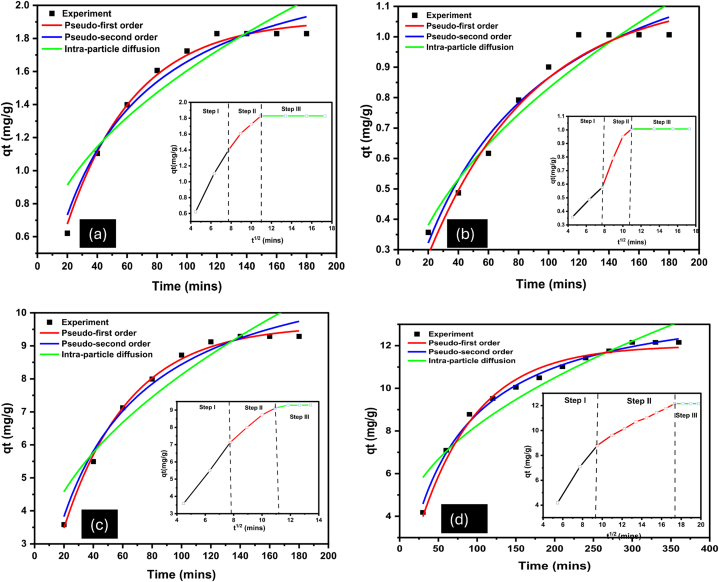
Non-linear models for the adsorption of Pb(II) onto (a) kaolin (b) organokaolin (c) bentonite and (d) organobentonite at room temperature.

The pseudo-first order, pseudo second order and the intra particle diffusion models were applied to understand the kinetics of the adsorption of Pb(II) onto the clay adsorbents. The non-linear kinetic model graphs and model parameters are shown in Fig. 5 and Table 7, respectively.

**Table 7:** Non-linear kinetic model parameters at room temperature.

Model Parameter	Adsorbent			
	Bentonite	Organobentonite	Kaolin	Organokaolin
	q_e_ (mg/g) = 10.742	14.960	1.830	1.007
Pseudo-first order				
q_m_ (mg/g)	9.624	12.012	1.916	1.139
K_1_ (1/min)	0.0186	0.0134	0.0218	0.0142
R^2^	0.9960	0.9839	0.9926	0.9700
RMSE	0.3605	7.741	0.1025	0.1211
χ^2^	0.0186	0.0601	0.0015	0.0021
Pseudo-second order				
q_m_ (mg/g)	12.078	16.565	2.430	1.496
K_2_ (g/mg × min)	0.00192	0.00105	0.00174	0.00196
R^2^	0.9903	0.9933	0.9708	0.9648
RMSE	4.929	0.6569	0.2043	0.1341
χ^2^	0.02432	0.04316	0.00596	0.00257
Intra-particle diffusion				
K_id_(mg/g.min^1/2^)	0.64143	0.0532	0.1252	0.0815
C	1.7094	2.8607	0.3529	0.01625
R^2^	0.9658	0.9116	0.9654	0.9352
RMSE	10.3039	2.3850	1.8455	0.1817
χ^2^	0.1064	0.5691	0.00341	0.00471

Based on the R^2^, RMSE and χ^2^ values obtained, it can be observed that the adsorption of Pb(II) onto kaolin, bentonite and organokaolin followed the pseudo-first order model. The pseudo-first order model assumes physisorption as the rate-limiting step [72]. This finding is supported by the isotherm results in the discussion in section 3.3. The values of q_m_ (calculated adsorption capacity) for pseudo-first order also depicted a better fitness of the model since they were closer to the q_e_ (experimental adsorption capacity) values. The adsorption of Pb(II) onto organobentonite followed the pseudo-second order model, as can be seen by the high R^2^ (0.9933) and low RMSE(0.6569) and χ^2^ (0.04316) values. Pseudo-second order model assumes chemisorption as the rate limiting step [25,72]. These results are also consistent with the isotherms results reported. The mean free-energy of adsorption calculated indicate chemisorption as the possible major mechanism of Pb(II) adsorption onto organobentonite. Moreover, the pseudo-second order q_e_ and q_m_ are closer together confirming this model better describes the adsorption data [25,69,73].

The two models cannot fully explain the origin of the mass transfer of Pb(II) ions onto the adsorbent's surface. Therefore, the intra-particle diffusion model was applied. The correlation coefficients for intra-particle diffusion were lower than those of the pseudo-first order and pseudo-second order models, indicating that diffusion of Pb(II) into the pores of the adsorbents is not the main adsorption mechanism. However, the C value obtained from the intra-particle diffusion model is not zero, suggesting that the adsorption of Pb(II) onto the adsorbents follows different mechanisms [25]. As shown in the plots of q_t_ versus *t*^1/2^ in Fig. 3(a–d), the removal of Pb(II) occurs in three stages. The first stage (Step I) shows the rapid diffusion of Pb(II) ions onto the clay's surface. The second stage (Step II) shows the gradual increase of Pb(II) adsorption before reaching equilibrium in the last stage (Step III). Similar observations were observed in other studies [25,74].

### 3.6 Thermodynamic studies

**Fig. 6:**
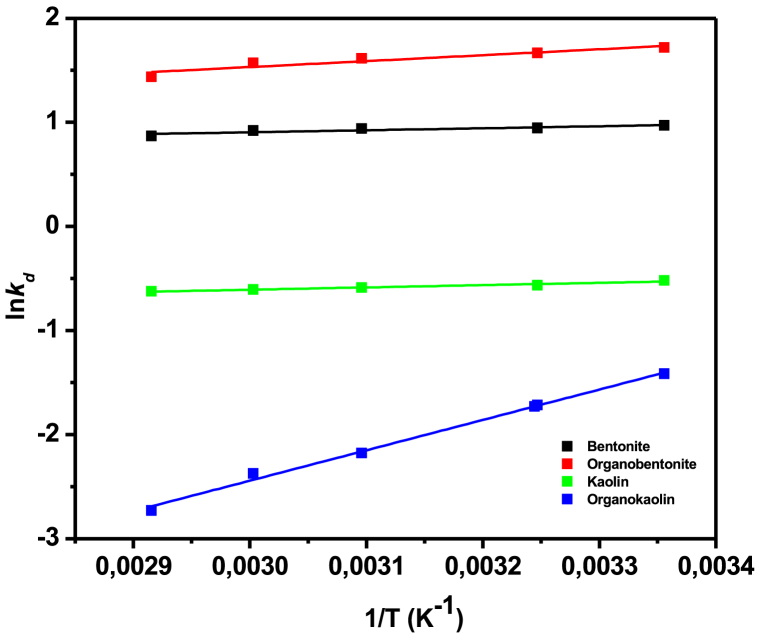
Thermodynamic plots for Pb(II) adsorption onto the four clay adsorbents.

To determine the thermodynamic parameters of the adsorption processes, the Van't Hoff equation was used to find the enthalpy change (ΔH), change in entropy (ΔS) and the Gibbs free-energy (ΔG) [25]. Fig. 6 and Table 8 show the plots of lnKd against 1/T, and the calculated thermodynamic parameters, respectively.

**Table 8:** Thermodynamic parameters for Pb(II) adsorption onto the four adsorbents.

Sorbent	ΔH^o^ (kJ/mol)	ΔS^o^ (J/mol.K)	ΔG^o^ (kJ/mol)				
			298 K	308 K	323 K	333 K	343 K
Bentonite y = 191.3975x–0.3308	−1.59	2.57	−2.40	−2.44	−2.48	−2.51	−2.53
Organobentonite y = 569.2723x-0.1752	−4.73	−1.47	−4.29	−4.28	−4.26	−4.24	−4.24
Kaolin y = 221.2933x-1.2718	−1.84	−10.57	1.31	1.42	1.57	1.68	1.79
Organokaolin y = 2918.87x-11.1993	−24.27	−93.11	3.48	4.41	5.80	6.74	7.67

The linear equations obtained from Fig. 6 as well as the thermodynamic parameters of the study are reported in Table 8. The enthalpy change (ΔH) was negative for all reactions, indicating the adsorption of Pb(II) onto clay adsorbents is an exothermic process [25,40]. The entropy change was positive for Pb(II) adsorption onto bentonite, indicating an increase in disorder within the reaction system. In contrast, kaolin, organokaolin, and organobentonite showed negative entropy changes, suggesting a stable arrangement of the adsorbate on the adsorbent surface [25].

Modifying bentonite with HDTMA alters its surface properties, including adsorption sites and surface charge, which affect the entropy change during Pb(II) adsorption. The surfactant disrupts the hydration structure around the clay, reducing the disorder of water molecules, resulting in a lower entropy for modified clays [75], i.e., bentonite (−1.47 J/mol.K) compared to natural bentonite (2.57 J/mol.K); and kaolin (−10.57 J/mol.K) compared to organokaolin (−93.11 J/mol.K). The negative ΔH and the increase in ΔG with temperature indicates that the reactions are less favorable at higher temperatures [76]. Based on these findings, it can be deduced that temperature increase does not improve the adsorption efficiency, thus, it can be concluded that although thermodynamics bring understanding regarding the behaviour of these adsorption processes, it is other factors such as specific surface area, pH, affinity of Pb(II) to the adsorbents and kinetics that play crucial roles in determining the efficiency of these adsorption processes [41].

### 3.7 Probable adsorption mechanisms

Ion-exchange is likely the primary mechanism of adsorption for Pb(II) ions onto natural bentonite. Bentonite's high CEC facilitates the exchange of cations such as Na^+^ and K^+^ in the interlayer spaces with the Pb(II) ions in solution. Surface complexation is thought to be the secondary mode of adsorption. Two forms of surface complexation, the first one involving the formation of outer-sphere complexes between Pb(II) ions and the permanent negative charges on the clay surface, and the second being inner-sphere complexeation with aluminol and silanol groups at the clay edges are other potentially involved adsorption mechanisms. When bentonite is modified with HDTMA, additional complexation with the organic groups may occur, particularly with the amine group introduced by HDTMA [77], leading to stronger interactions than those with unmodified bentonite, as indicated by the reported E values. The expansion of interlayer spacing in organobentonite also allows for the possible intercalation of Pb(II) ions between clay layers [78,79], either through ion exchange or surface complexation with HDTMA molecules. However, studies on the point of zero charge and adsorption pH for the modified clay exclude ion exchange as the major removal mechanism of Pb(II) onto organobentonite. These additional adsorption mechanisms in organobentonite may explain its increased Pb(II) adsorption capacity compared to natural bentonite.

Similar mechanisms apply to the adsorption of Pb(II) onto natural kaolin and organokaolin, though kaolin's lower CEC and limited interlayer spacing expansion make intercalation less likely. The introduction of HDTMA into kaolin also significantly reduces its hydrophilic nature, making it difficult for the clay to mix with the Pb(II)-containing solution. As a result, the adsorption performance is decreased. These findings highlight the complexity of Pb(II) adsorption mechanisms across the natural clays and their modified forms.

### 3.8 Real water treatment and reusability studies

**Fig. 7:**
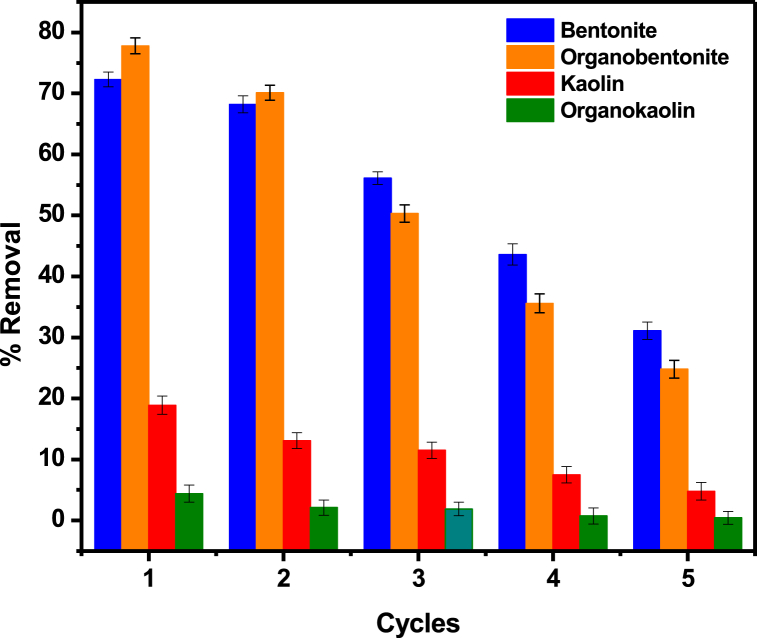
Reusability cycles of the four adsorbents on Pb(II) uptake.

The application of the clay adsorbents to real water samples is of crucial importance for industrial application. Fig. 7 shows the treatment of borehole water samples using the four adsorbents. The removal percentages for Pb(II) ions in solution was 72.3 % for bentonite, 77.8 % for organobentonite, 21.9 % for kaolin and 5.4 % for organokaolin. Due to the loss of the clay sample with each regeneration cycle, 1.0 g mass was used as the initial mass for this study. Considering the initial mass used in this experiment was 1.0 g, compared to the mass of 200 mg used for the simulated solutions, it is evident that the percentage removal for Pb(II) in borehole water samples is lower compared to that of simulated solutions. This can be attributed to the presence of other competing ions in solution. The real water sample contains trace amounts of other PTEs such as Cd, Cr, As and others, which all compete for the active sites on the adsorbents.

The reusability of adsorbents is another important factor in determining the economic feasibility of the adsorbents in terms of production costs. Therefore, investigating the reusability of adsorbent materials is essential. The spent adsorbents were regenerated using 0.01 M HCl as a desorbing agent [37]. Fig. 7 shows that the removal efficiency of the adsorbents decreased with each of the four regeneration cycles. For the first two cycles, organobentonite removed more Pb(II) ions from solution compared to the natural bentonite, however, from cycle 3 the performance of organobentonite decreased sharply and the regenerated natural bentonite showed greater efficiency than that of organobentonite. The regeneration of organobentonite with HCl may lead to the destruction of the surfactant, and therefore changing the chemistry of the clay. This results in decreased performance of the clay with each regeneration cycle [80]. The decrease in Pb(II) percentage removal with each cycle may also be due to incomplete Pb(II) ions removal from previous cycles, which reduce the available active sites for the adsorption of more Pb(II) ions [37]. The successful regeneration of the adsorbents for up to four cycles indicate their potential as a stable and re-useable adsorbent for Pb(II) removal from real water samples.

## 4 Conclusion

In this study, organokaolin and organobentonite were successfully synthesized using HDTMA as the modifier, and the adsorbents’ efficiency in removing Pb(II) from aqueous solutions was investigated. Organobentonite showed a higher Pb(II) adsorption capacity (18.75 mg/g) compared to the natural bentonite (14.71 mg/g), while the opposite effect was observed for kaolin (4.19 mg/g) and organokaolin (2.26 mg/g). The most favorable conditions for Pb(II) removal were pH = 6, room temperature (23–25 °C) and adsorbent mass of 200 mg. The ideal contact time for adsorption by organokaolin, kaolin and bentonite were 120 min, while for organobentonite it was 300 min. This time increase in organobentonite-Pb(II) adsorption is due to the formation of new chemical bonds. The Langmuir isotherm and pseudo-first order kinetic models governed the adsorption properties of Pb(II) onto kaolin, organokaolin and bentonite; while the Freudlich and pseudo-second order governed the adsorption of Pb(II) onto organobentonite. The adsorption reactions were all exothermic, and an increase in temperature proved not to improve the adsorption capacity. The results of the removal of Pb(II) using kaolin and organokaolin were unsatisfactory due to the low adsorption capacities determined. The combination of bentonite and HDTMA did however demonstrate the synergetic advantage towards Pb(II) adsorption. The reusability study also revealed that the spent adsorbents can be reused to achieve good Pb(II) percentage removal for up to four cycles. Overall, the results of this study showed that organobentonite and bentonite can serve as promising materials for the removal of Pb(II) ions from water/wastewater in South Africa and other countries. However, modifying bentonite with HDTMA increases the costs of the adsorbent material. The unmodified natural bentonite yielded about 79 % of the capacity of removal of Pb(II) by organobentonite. Furthermore, to achieve the best removal of Pb(II) by organobentonite, it required 300 min compared to the contact time of 120 min, which needed by bentonite to achieve the highest removal of Pb(II). Thus, taking into consideration costs incurred by modification and the longer time required to achieve the best removal of Pb(II) using organobentonite as an adsorbent material, authors recommend the use of natural bentonite, which yielded quantitative removal of Pb(II) from water. This study is not only limited to Pb but can also be extended to treating other PTEs such as Cr, As, Cd and others from aqueous solutions.

## Data availability statement

Data will be made available on request.

## CRediT authorship contribution statement

**Dipuo Precious Kgabi:** Writing – original draft, Visualization, Validation, Methodology, Investigation, Formal analysis, Data curation, Conceptualization. **Abayneh Ataro Ambushe:** Writing – review & editing, Visualization, Validation, Supervision, Resources, Project administration, Methodology, Investigation, Funding acquisition, Conceptualization.

## Declaration of competing interest

The authors declare the following financial interests/personal relationships which may be considered as potential competing interests:Abayneh Ataro Ambushe reports financial support was provided by 10.13039/501100004424Water Research Commission. Dipuo Precious Kgabi reports financial support was provided by 10.13039/501100001321National Research Foundation. If there are other authors, they declare that they have no known competing financial interests or personal relationships that could have appeared to influence the work reported in this paper.
